# Antibacterial activity of ethanolic extract and compounds from fruits of *Tectona grandis* (Verbenaceae)

**DOI:** 10.1186/s12906-015-0790-5

**Published:** 2015-08-06

**Authors:** Gabin Thierry M. Bitchagno, Leonard Sama Fonkeng, Théodora K. Kopa, Michel F. Tala, Hippolyte Kamdem Wabo, Christopher B. Tume, Pierre Tane, Jules-Roger Kuiate

**Affiliations:** Department of Chemistry, University of Dschang, P.O. Box 67, Dschang, Cameroon; Department of Biochemistry, University of Dschang, P.O. Box 67, Dschang, Cameroon; Institute of Medical Research and Medicinal Plants Studies (IMPM), P.O. Box 6163, Yaounde, Cameroon

**Keywords:** *Tectona grandis*, Fruits, Quinones, Terpenoids, Antibacterial

## Abstract

**Background:**

Well known as teak, *Tectona grandis* is widely used in African folk medicine for its pharmacological relevance. In Cameroon, this species is a reputed laxative in the Northern Region while in the Western Region, it is used in the treatment of skin diseases and diarrhoea.

**Materials and methods:**

Separation and isolation of compounds were performed using different chromatographic methods while their structures were elucidated by spectroscopic techniques including MS and NMR, and by comparison of data with those reported in the literature. Isolated compounds as well as crude ethanol extract were tested for their antibacterial activities using broth micro-dilution method against four Gram negative bacteria strains *Escherichia coli* (ATCC 8739), *Pseudomonas aeruginosa* (PA 01), *Klebsiella pneumonia* (ATCC 11296) and *Escherichia aerogenes* (ATCC 13048).

**Results:**

Three known compounds were isolated, including two quinones and one triterpene. They were identified as tectograndone (**1**), 6-methyl-1,4-dihydroxyanthraquinone (**2**), and 2β-hydroxyursolic acid (**3**) respectively. Crude ethanol extract showed good activity against the bacteria strains tested with MIC of 64–256 μg/mL. Among the isolated metabolites, 6-methyl-1,4-dihydroxyanthraquinone exhibited a strong activity against *Escherichia aerogenes* with MIC of 16 μg/mL, while tectograndone showed a moderate activity against *Escherichia coli* with MIC of 32 μg/mL. The antibacterial screening of the fruits of this plant as well as that of compounds **1** and **2** is reported herein for the first time.

**Conclusion:**

The research work presented here shows that *Tectona grandis* fruits possess compounds which could be developed in the treatment of bacterial diseases.

## Background

*Tectona grandis* Linn. is a large tree from Southeast Asia which grows up to 50 m in height. It is the most important species of the genus *Tectona* [1] and has been naturalized in Africa. Well known as teak, *T. grandis* is widely reputed for its strong and straight trunk which is used in the construction industry. Traditionally, this species is globally used to relieve fever, diabetes, lipid disorders, ulcers, inflammation, bronchitis, cancer and tuberculosis [2, 3]. In the Northern part of Cameroon, leaves of teak are used for their laxative properties while in the Western Region, it is used for the treatment of skin diseases. Previous phytochemical investigation of *Tectona* species have led to the isolation of triterpenoids, flavonoïds [4], chromomoric acid derivatives [5], anthraquinones [6–8], naphthoquinones [9, 10], anthraquinone-naphthoquinones [11, 10], apocarotenoids [1] and lignans [12]. Some of these metabolites particularly the quinines showed antimycobacterial, antifungal and allelopathic activities [7, 9, 11, 1, 10]. In the course of our ongoing search for potent bioactive compounds from Cameroonian medicinal plants [6, 13, 14], we carried out the chemical investigation of the fruit of *T. grandis* and report herein the antibacterial properties of compounds isolated from the ethanolic extract.

## Methods

### General experimental procedures

Melting points of the isolated compounds were determined using an Electrothermal IA9000 Series digital melting point apparatus (Bibby Scientific, Great Britain). MS detection was carried out using a Waters Micromass ESI-Q-TOF II instrument with ESI ionization in the positive mode. EIMS spectra were recorded on a Finnigan MAT 95 spectrometer (70 eV) with perfluorokerosine as reference substance for HR-ESI-TOF-MS (Japan). IR spectra were recorded on a Shimadzu FTIR-8400S spectrophotometer (Japan). UV spectra were recorded on a Shimadzu UV-160A spectrometer (Japan) in absolute ethanol and alkaline ethanol. The NMR spectra were measured on Bruker 500 MHz NMR Avance II spectrometer equipped with cryoprobe, with TMS as an internal reference. Chemical shifts were recorded in δ (ppm) and the coupling constants (*J*) are in hertz (Hz). Silica gel 60 *F*_254_ (70–230; Merck; Darmstadt, Germany) was used for column chromatography. Precoated silica gel Kieselgel 60 *F*_254_ plates (0.25 mm thick) were used for TLC, and spots detected by spraying with 50 % H_2_SO_4_ followed by heating at 100 °C.

### Plant material

The fruits of *T. grandis* were collected in Banyo, Adamaoua Region of Cameroon in January 2011. The species was identified at the Cameroon National Herbarium (Yaoundé), by comparing with a voucher specimen No. 61993 HNC.

### Extraction and fractionation

Dried fruits of *Tectona grandis* (2.5 Kg) were extracted with ethanol (10 L) for 72 h at room temperature to yield a crude extract (55 g) after evaporation under reduced pressure. This extract (50 g) was subjected to silica gel column chromatography eluted with gradients of *n*-hexane-EtOAc and EtOAc-MeOH. Ninety fractions of 300 mL each were collected using mixtures of n-hexane-EtOAc 85:15, 70:30, 30:70 and combined on the basis of their TLC profiles into four main fractions coded A-D (A: 1–19; B: 20–46; C: 47–68; D: 69–90). Fraction A (20 g) contained mostly fatty material and was not further investigated. Fraction B (6.5 g) was separated by a column chromatography over silica gel using a gradient of *n*-hexane-EtOAc (100:0, 95:5, 90:10, 85:15, 80:20, 75:25 and 70:30) to afford five sub-fractions (FrB1-FrB5). Following their TLC profiles, only FrB3 was retained for further purification over silica gel column chromatography with *n*-hexane-EtOAc to afford 2β-hydroxyursolic acid (**3**) (10 mg). Fraction C (10 g) was subjected to column chromatography over silica gel eluted with *n*-hexane-EtOAc (90:10, 85:15, 80:20, 75:25 and 70:30). The collected fractions which contained the major compound 6-methyl-1,4-dihydroxyanthraquinone (**2**) were combined and applied on a Sephadex LH-20 column (*n*-hexane-dichloromethane-methanol, 7:4:0.5) to give 8 mg while the remaining complex material was kept aside for further investigation. Similary, repeated column chromatography of fraction D (2 g) yielded tectograndone (**1**) (8 mg).

Tectograndone (**1**): Red powder in acetone, ^13^C NMR (CDCl_3_- DMSO-*d*_*6*_, 125 MHz) δ (ppm) : 187.5 (C-1’), 185.5 (C-6), 183.8 (C-11), 188.2 (C-4’), 157.9 (C-8’), 157.2 (C-5’), 155.8 (C-5), 150.7 (C-12a), 149.0 (C-12), 145.7 (C-8), 138.4 (C-9), 135.6 (C-2’), 132.4 (C-10), 132.3 (C-3), 131.5 (C-6’), 130.2 (C-10a), 128.9 (C-7), 128.1 (C-7’), 126.6 (C-3’), 125.8 (C-6a), 117.0 (C-4a), 115.7 (C-4), 113.4 (C-11a), 111.6 (C-4a’), 79.8 (C-2).

6-methyl-1,4-dihydroxyanthraquinone (**2**) : Red powder in acetone, ^13^C NMR (DMSO-*d*_*6*_, 125 MHz) δ (ppm) : 186.6 (C-9), 186.3 (C-10), 156.6 (C-1), 156.6 (C-4), 146.0 (C-6), 135.6 (C-7), 132.6 (C-5a), 130.4 (C-8a), 129.2 (C-2), 129.1 (C-3), 126.6 (C-5), 126.6 (C-8), 112.6 (C-4a), 112.4 (C-9a).

2β-hydroxyursolic acid (**3**) :White powder in MeOH, ^13^C NMR (DMSO-*d*_*6*_, 125 MHz) δ (ppm) : 178.1 (C-28); 138.2 (C-13); 124.4 (C-12); 79.0 (C-3); 64.5 (C-2); 52.3 (C-5); 47.5 (C-18); 46.8 (C-1); 46.7 (C-17); 41.6 (C-9); 40.1 (C-14); 39.9 (C-4); 38.4 (C-8); 38.3 (C-20); 37.8 (C-19); 37.6 (C-10); 36.2 (C-22); 32.5 (C-7); 30.1 (C-21); 28.7 (C-23); 27.3 (C-15); 23.7 (C-16); 23.2 (C-27, C-11); 22.8 (C-30); 20.9 (C-6); 17.5 (C-24); 16.8 (C-26, C-29); 16.4 (C-25).

The qualitative analysis of the ethanol extract of the teak fruit was also conducted by using the method described by Harbone (1973) [15] with slight modifications.

### Antibacterial assay

#### Microorganisms

Microorganisms used in this study were four Gram-negative bacteria strains *Escherichia coli* (ATCC 8739), *Pseudomonas aeruginosa* (PA 01), *Klebsiella pneumonia* (ATCC 11296) and *Escherichia aerogenes* (ATCC 13048) all of which were reference strains obtained from the American Type Culture Collection. The bacterial strains were grown at 35 °C and maintained on Mueller Hinton Agar (MHA) (Titan Biotech Ltd Rajasthan India).

### Preparation of inoculum

The inoculum was prepared as described by Tereshuck et al. [16] from 24 h old cultures by picking numerous colonies and suspending them in sterile saline (NaCl, 0.9 %) solution. Absorbance was read at 530 nm and adjusted with the saline solution to match to that of a 0.5 McFarland standard solution, corresponding to about 1.5 × 10^8^ Colony Forming Units (CFU).

### Preparation of extract and determination of Minimum Inhibitory Concentrations (MICs) and Minimum Bactericidal Concentration (MBCs)

The antibacterial activity was investigated by determining the minimum inhibitory concentrations (MICs) and the minimum bactericidal concentrations (MBCs). MICs were determined by a broth micro-dilution method with slight modification of the method described by Newton et al. (2012) [17].

Stock solutions of the extract and compounds were prepared in the Mueller Hinton Broth (MHB) (Titan Biotech Ltd Rajasthan India) in 5 % (v⁄v) dimethylsulfoxide (DMSO) solution (Fisher chemicals, Strasbourg, France) for a final concentration of 4096 μg/mL and 1024 μg/mL respectively for extract and compounds.

Into each well of 96-microplate (Nunclon, Roskilde, Denmark) 100 μL of MHB and 100 μL of the test substance solution were introduced. Twofold were serial dilutions was made to obtain a concentration range of 8–1024 μg/mL for crude extract and 8–256 μg/mL compounds. Bacterial inoculums (400 μL) prepared above was added to MHB (15 mL) for a final concentration of 4 × 10^6^ CFU/mL which was used for this test. One hundred microliters of this inoculum was introduced to each well containing 100 μL of MHB and extract mixture to a final volume of 200 μL. The final concentration of DMSO in the well was less than 1 % (preliminary analyses show that 1 % (v/v) DMSO does not inhibit the growth of the test organisms). A sterility check (5 % DMSO, media, inoculum and water soluble antibiotic) was included in the experiment. The plates were covered with a sterile lid, and incubated at 35 °C for 24 h under shaking using a plate shaker (Flow Laboratory Germany) at 300 rpm. After this incubation, the MICs were assessed by adding 40 μL of 2 % solution of p-iodonitrotétrazolium (INT) (Sigma-Aldrich, South Africa) in each well. Viable bacteria cause the appearance of pink coloration in the presence of this solution [18]. The concentration that did not show the appearance of pink solution was considered as the inhibition concentration and the smallest one was noted as the MIC. For the well that did not present color changes, 50 μL aliquots of solution of the corresponding well which did not receive INT were put into the well of a new plate containing 150 μL of freshly prepared MHB and re-incubated at 35 °C for 48 h on the shaker. After this re-incubation, 40 μL of INT were introduced in each well and all the concentrations that did not present color change were considered as the bactericidal concentration and the smallest one was noted as MBC. The assay was repeated thrice. Ciprofloxacine at the concentration range of 0.039-5 μg/mL served as positive control.

## Results

**Fig. 1:**
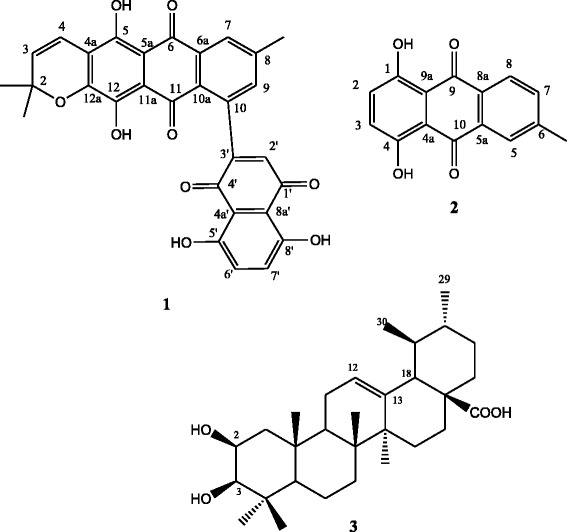
Chemical structures of compounds 1–3

The structures of the isolated compounds were elucidated using modern spectroscopic methods (IR, ^1^H and ^13^C NMR, HRMS and 2D-NMR). Comparison of data with those reported in the literature led to the identification of the compounds as tectograndone (**1**) [11], 2β-hydroxyursolic acid (**3**) [19] and 6-methyl-1,4-dihydroxyanthraquinone (**2**) [20] (Fig. 1). Previous phytochemical investigation of teak leaves also led to the isolation of triterpenoids [4], anthraquinones [6–8] and naphthoquinones [9, 10].

### Phytochemical analysis

In this study, the phytochemical composition of the fruits extract of *T. grandis* was evaluated and we investigated the antibacterial activity of extract together with isolated compounds investigated. Qualitatively, the crude extract contains anthraquinones, polyphenols, sterols, triterpenes and tannins but not flavonoïds, xanthocyanates and saponins (Table 1).

**Table 1:** Phytochemical classes of compounds in the ethanol extract of the fruits of *T. grandis*

	Flavonoid	Anthraquinone	Polyphenol	Sterol	Triterpen	Xanthocyanate	Tanin	Saponin
Crude extract	-	+	+	+	+	-	+	-

(−)absent; (+) present

Moreover, the results shown in Table 2 demonstrate that the ethanol extract of teak fruit displayed good activities against *Escherichia coli* (ATCC 8739), *Pseudomonas aeruginosa* (PA 01), *Klebsiella pneumonia* (ATCC 11296) and *Escherichia aerogenes* (ATCC 13048) with the MIC values ranging from 64 to 256 μg/mL. The isolated compounds(**1**–**3**) showed potent antibacterial activity toward these microorganisms. This supports the use of this plant in traditional medicine in the treatment of skin diseases. Compounds **1** and **2**, with the same basic quinoidal skeletal units, were the most active against *E. coli* (32 μg/mL) and *E. aerogene* (16 μg/mL) respectively. In addition, compound **2** also presented a considerable antibacterial activity against *P. aeruginosa* (128 μg/mL).

**Table 2:** Minimum inhibitory concentration and minimum bactericidal concentration (μg/mL) of the ethanol extract and compounds of the fruits of *T. grandis*

Bacteria strains	Parameters	EtOH extract	Tectograndone (**1**)	6-methyl-1,4-dihydroxyanthraquinone (**2**)	2β-hydroxyursolic acid (**3**)	Ciprofloxacine^a^
*Escherichia coli* ATCC8739	MIC	64	32	>256	128	5
	MBC	128	128	>256	256	5
	MBC/MIC	2	4	-	2	1
*Pseudomonas aeruginosa* PA01	MIC	256	>256	128	64	0.0625
	MBC	256	>256	256	128	0.0625
	MBC/MIC	1	-	2	2	1
*Klebsiella pneumonia* ATCC1148	MIC	128	>256	>256	64	5
	MBC		>256	>256	128	5
	MBC/MIC	256 2	-	-	2	1
*Escherichia aerogenes* ATCC13048	MIC	64	>256	16	64	2.5
	MBC	128	>256	128	128	2.5
	MBC/MIC	2	-	8	2	1

^a^Reference drug

## Discussion

It has been reported that the number and position of hydroxyl groups in phenolic compounds such as anthraquinones can significantly influence their antimircobial activity [14]. Generally, those possessing free hydroxyl group(s) display good activities. Some examples include physcion, emodin, and fallacinol [21, 22, 14]. Compound **3** showed a moderate antibacterial activity (64–128 μg/mL) against all tested bacteria. Previously, it had been shown that ursolic acid, a compound structurally related to **3** also exhibited moderate activity against *E. coli* but none toward *P. aeruginosa* [23]. All these isolated compounds (**1**, **2**, **3**) showed antibacterial activity on at least one strain of bacteria together while the crude extract which presented high activity against all the bacteria strains. Some individual anthraquinones, tannins, polyphenols, sterols and triterpenes have shown similar types of biological activities [24–30]. Their synergic effect was also reported [31].

Our data showed that the response of the bacteria to the tested compounds varied from one microorganism to another. This difference in susceptibility may be explained by the difference in cell wall composition and/or genetic content of plasmids that can be easily transferred amount microbial strains [32]. It was also found that MBC values obtained were generally less than fourfold of their MICs values (Table 2) on the bacteria species. This suggests that a bacteriocidal effect of the crude extract and the isolated compounds could be expected on most the tested bacteria [33, 34]. This is interesting in view of the prospect of developing new antibacterial drugs from the tested samples. To the best of our knowledge, this is the first report on the antibacterial activity of the crude extract and compounds from fruit of *T. grandis*.

The overall results of this study can be considered promising in view of the need to develop of new phytodrugs for the fight against bacterial infections of public health importance. *P. aeruginosa* has emerged as one of the most problematic Gram-negative pathogens, with an alarmingly high antibiotic resistance rate [35, 36]. Even with the most effective antibiotics against this pathogen, namely the carbapenems (imipenem and meropenem), the level of resistance was found to be about 15–20.4 % among the 152 tested *P. aeruginosa* strains [36]. This pathogen was found to be sensitive to the crude extract and two of the isolated compounds (**1**, **2**).

## Conclusion

The results of the present study provide an important basis for use of the ethanol extract from the fruits of *T. grandis* for the treatment of skin diseases. The crude extract as well as the isolated compounds found to be active in this study could also be useful for the development of new antibacterial drugs. However, further pharmacological and toxicity studies currently going on in our laboratory will be necessary to establish if they could be safely used as topical antibacterial agents.
